# Characteristics of *NRAS*-mutated patients with chronic myelomonocytic leukemia in a national (ABCMML) and an international cohort (cBioPortal)

**DOI:** 10.1007/s10354-025-01080-0

**Published:** 2025-05-15

**Authors:** Alexandra Qian, Klaus Geissler

**Affiliations:** 1 Medical School, Sigmund Freud University, Sigmund Freud Platz 3, 1020 Vienna, Austria; 2 Karl Landsteiner Institut für hämatoonkologische Forschung, St. Pölten, Austria

**Keywords:** CMML, NRAS, Austrian biodatabase for chronic myelomonocytic leukemia, cBioPortal, Mutations, CMML, NRAS, „Austrian biodatabase for chronic myelomonocytic leukemia“, cBioPortal-Datensatz, Mutationen

## Abstract

Big data from large international cooperations allow proving consistency of findings in national patient cohorts. We compared features in *NRAS*-mutated patients of the Austrian biodatabase for chronic myelomonocytic leukemia (ABCMML) with the chronic myelomonocytic leukemia (CMML) cohort documented in cBioPortal. It is consistently demonstrated in both CMML cohorts that *NRAS* mutations are associated with shorter overall survival, acute myeloid leukemia-free survival, increased white blood cell count, decreased platelet values and the presence of blast cells in peripheral blood. Hemoglobin values did not differ between patients with or without *NRAS* mutations in either cohort. Validation of data in CMML patients using two independent cohorts, ensures the quality standards for clinical decision making.

**Supplementary Information:**

The online version of this article (10.1007/s10354-025-01080-0) contains supplementary material, which is available to authorized users.

## Abstract (de)

Große Datenmengen aus internationalen Kooperationen ermöglichen die Überprüfung der Datenkonsistenz nationaler Patientenkohorten. Wir verglichen die Ergebnisse von Patienten mit *NRAS*-Mutation aus der nationalen „Austrian biodatabase for chronic myelomonocytic leukemia (ABCMML)“ mit jenen von Patienten, die in der internationalen Plattform des cBioPortal dokumentiert sind. Eine Konsistenz der Ergebnisse in beiden Patientenkohorten fand sich im Hinblick auf ein schlechteres Gesamtüberleben und auf ein kürzeres, von akuter myeloischer Leukämie freies Überleben bei Patienten mit *NRAS*-Mutation. Höhere Leukozytenzahlen, geringere Thrombozytenwerte und ein häufigeres Vorkommen zirkulierender Blasten bei Patienten mit *NRAS*-Mutation waren ebenfalls in beiden Kohorten nachweisbar. In keiner der beiden Gruppen unterschieden sich die Hämoglobinwerte der Patienten mit *NRAS*-Mutation von jenen ohne *NRAS*-Mutation. Die Validierung von Daten in zwei unabhängigen Kohorten stellen die erforderlichen Qualitätsstandards für klinische Entscheidungsfindungen sicher.

## Introduction

Chronic myelomonocytic leukemia (CMML) is a rare, genotypically and phenotypically heterogenous hematologic malignancy of elderly people with an intrinsic risk of progression and transformation into secondary acute myeloid leukemia (AML). With regard to the presence of myeloproliferation, CMML was originally subdivided into myeloproliferative disorder (MP-CMML; WBC count > 13 × 10^9^/L) versus myelodysplastic syndrome (MD-CMML; WBC count ≥ 13 × 10^9^/L) by the French-American-British (FAB) criteria [1, 2]. Since CMML is characterized by features of both myelodysplastic syndrome (MDS) and myeloproliferative neoplasm (MPN), the World Health Organization (WHO) classification of 2002 assigned CMML to the mixed category (MDS/MPN) [3]. After the 2016 revision of the World Health Organization classification of myeloid neoplasms and acute leukemia [4], updated diagnostic criteria for CMML were reported recently by two groups [5, 6]. CMML patients may have highly variable outcomes, suggesting that several factors can determine the course of disease and the causes of death in these patients [7–13].

Recently, we reported on the Austrian biodatabase for CMML (ABCMML). In the ABCMML, epidemiologic, hematologic, biochemical, clinical, immunophenotypic, cytogenetic, molecular and biologic data of patients with CMML have been collected from different Austrian centers for 40 years [14]. It has been shown to be a representative and useful real-life data source for biomedical research.

Due to the molecular heterogeneity of CMML, it is important to know the meaning of molecular characteristics in order to offer the patient the best possible management of their individual situation. Some studies have analyzed the impact of molecular aberrations on the clinical outcome and phenotype of disease, although findings in most studies were not validated by independent cohorts. However, a prognostic parameter should not enter clinical practice unless it has been demonstrated that it performs a useful role. External validation denotes evaluation of the performance of a prognostic parameter in a sample independent from that used to develop the model [15].

Big data containing a huge number of datasets from international large consortium efforts are now available for many cancer entities, including CMML. The cBioPortal platform is such a collection of big data aimed at building a platform in order to support clinical decisions for personalized cancer treatment [16]. Moreover, due to the large number of well-characterized patients, it is a perfect source of data for validation of findings in traditional, sometimes much smaller patient cohorts. In this study, we used data from CMML patients documented in cBioPortal to validate the features of *NRAS*-mutated CMML patients that have been analyzed in the ABCMML.

## Patients and methods

Patient data for this study were obtained from 2 sources as recently reported [17]

### ABCMML analysis

In the ABCMML database [13] we retrospectively collected epidemiologic, hematologic, biochemical, clinical, immunophenotypic, cytogenetic, molecular and biologic data of patients with CMML from different centers. The diagnosis of CMML and leukemic transformation were according to the WHO criteria [2–4]. Clinical and laboratory routine parameters were obtained from patient records. A detailed central manual retrospective chart review was carried out to ensure data quality before the analysis of data from institutions. CMML patients in transformation were not included in this study. In 327 patients, mutational data were available to analyze overall survival (OS), acute myeloid leukemia (AML)-free survival, and differences in phenotypic parameters between mutated and wildtype patients. This research was approved by the ethics committee of the City of Vienna on 10 June 2015 (ethic code: 15-059-VK).

### cBioPortal analysis

We selected from cBioPortal [16] the myelodysplastic syndromes [18] dataset containing 399 CMML cases with data including age, sex, white blood cell counts (WBC), hemoglobin (Hb), platelets, overall survival, AML-free survival, bone marrow (BM) blasts, circulating blasts, cytogenetics, and gene mutations (http://www.cbioportal.org) to analyze OS, AML-free survival, and differences in phenotypic parameters between mutated and non-mutated patients.

### Statistical analysis

The log-rank test was used to determine whether individual parameters were associated with OS. OS was defined as the time from sampling to death (uncensored) or last follow-up (censored). AML-free survival was defined as the time from sampling to transformation into AML or death (uncensored) or last follow-up (censored). Dichotomous variables were compared between different groups using the chi-square test. The Mann–Whitney U test was used to compare two unmatched groups when continuous variables were not normally distributed. Results were considered significant at *p* < 0.05. Statistical analyses were performed with SPSS v. 27 (SPSS Inc., Armonk, NY, USA); the reported *p*-values were two sided. In the ABCMML database, mutations with a variant allele frequency (VAF) of at least 5% and in the cBioPortal platform with a VAF of at least 2% are considered positive.

## Results

### Characteristics of patients and prevalences of *NRAS* mutations

The baseline characteristics of both CMML cohorts are shown in supplementary tables 1 and 2. A total of 327 patients in the ABCMML cohort and 399 patients in the cBioPortal cohort were analyzed. As seen in other CMML series, in both cohorts, there was a male predominance among CMML patients and more than half of the patients were aged 70 years or older [14]. All characteristics except leukocytes were comparable between the cohorts. The proportion of patients with leukocytes > 13 G/L was significantly higher in the ABCMML cohort as compared to the cBioPortal cohort (57% vs. 32%, *p* < 0.001). The median leukocyte counts were 14.1 vs. 9.2 G/L in these cohorts, respectively. Regarding clinical outcome, the median survival was 29.0 months in the ABCMML cohort as compared to 31.6 months in the cBioPortal cohort. The prevalences of *NRAS* mutations were 14.6% (46/316) in the ABCMML group and 15.7% (60/383) in the BIOPOPRTAL group, respectively.

### Impact of *NRAS* mutations on survival and AML-free survival

**Fig. 1:**
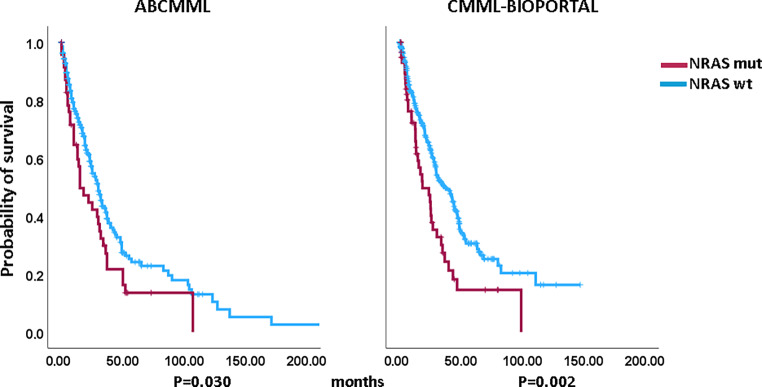
Kaplan–Meier plots for overall survival in chronic myelomonocytic leukemia (CMML) patients with and without *NRAS* mutations

**Fig. 2:**
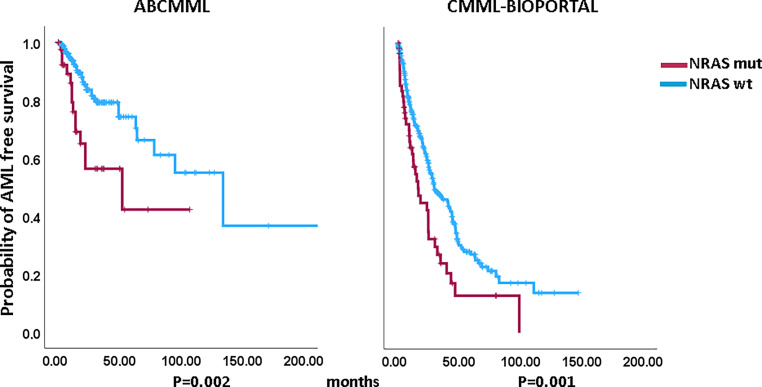
Kaplan–Meier plots for acute myeloid leukemia (AML)-free survival in chronic myelomonocytic leukemia (CMML) patients with and without *NRAS* mutations

Figures 1 and 2 show the Kaplan–Meier curves of OS in *NRAS*-mutated (variants and variant allele frequencies are shown in supplementary tables 3 and 4) and *NRAS*-nonmutated patients in both cohorts. In both cohorts, *NRAS*-mutated patients had significantly inferior survival. The median survival of *NRAS*-mutated patients was 15.0 vs. 30.0 months (*p* = 0.030) in the ABCMML patients and 18.5 vs. 36.3 (*p* = 0.002) months in the cBioPortal patients. Regarding AML-free survival, there was also a significant difference between *NRAS*-mutated and *NRAS*-nonmutated patients in both cohorts. The median AML-free survival was 52.0 vs. 134.0 (*p* = 0.002) months in the ABCMML cohort and 16.6 vs. 29.7 (*p* = 0.001) months, respectively, in the cBioPortal cohort.

### Laboratory features in the presence or absence of *NRAS* mutations

**Fig. 3:**
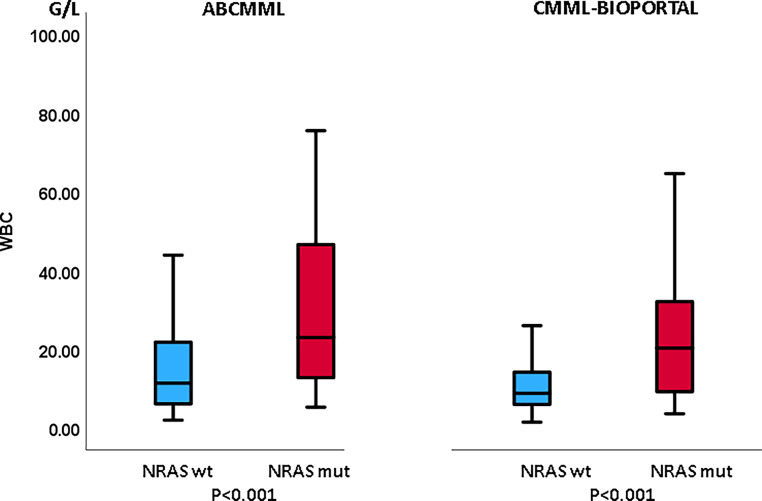
Boxplots showing the distribution of leukocytes in *NRAS*-nonmutated and *NRAS*-mutated chronic myelomonocytic leukemia (CMML) patients including median values, minimum values, maximum values, and upper and lower quartiles in both study cohorts. *WBC* white blood cell counts

**Fig. 4:**
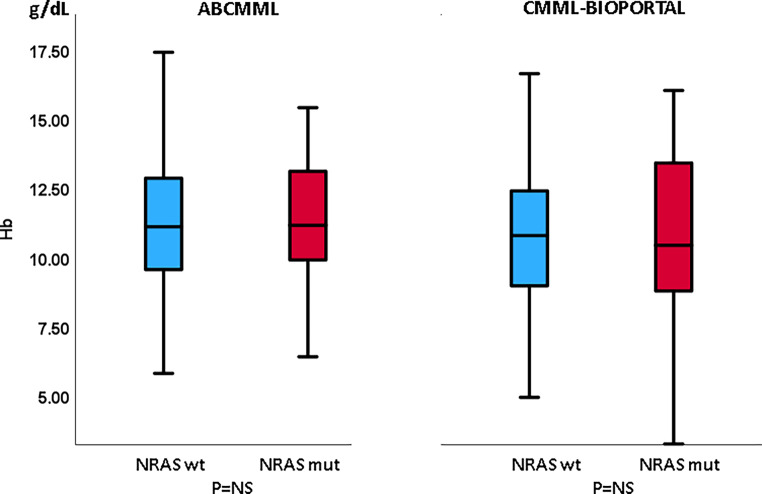
Boxplots showing the distribution of hemoglobin values in *NRAS*-nonmutated and *NRAS*-mutated chronic myelomonocytic leukemia (CMML) patients including median values, minimum values, maximum values, and upper and lower quartiles in both study cohorts. *Hb* hemoglobin

**Fig. 5:**
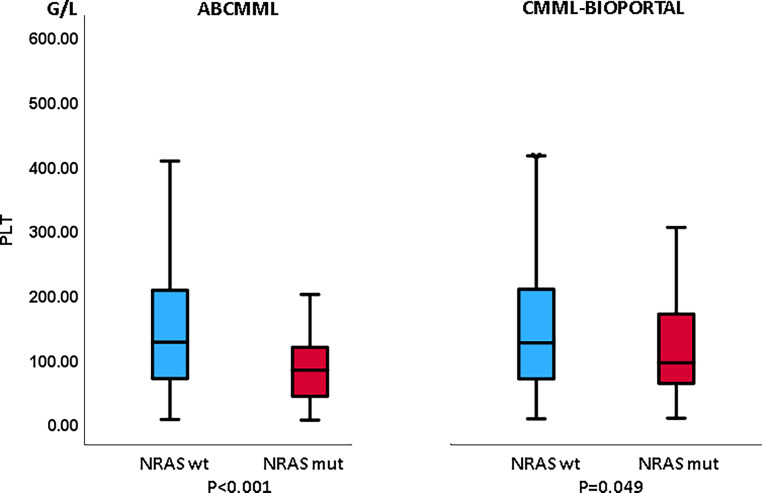
Boxplots showing the distribution of platelets in *NRAS*-nonmutated and *NRAS*-mutated chronic myelomonocytic leukemia (CMML) patients including median values, minimum values, maximum values, and upper and lower quartiles in both study cohorts. *PLT* platelet counts

Tables 1 and 2 show the phenotypic parameters in the ABCMML and cBioPortal patients, respectively. In both cohorts, *NRAS*-mutated patients had a significantly higher proportion of patients with leukocytosis > 13 G/L and patients with circulating blasts. Moreover, in both cohorts, *NRAS*-mutated patients had a significantly higher proportion of patients with thrombocytopenia, whereas the proportion of patients with anemia did not differ. In Figs. 3, 4 and 5, metric values are visualized by boxplot diagrams. In the ABCMML cohort, the median values of *NRAS*-mutated and nonmutated patients were for WBC 23.0 vs. 11.4 G/L, for Hb 11.1 vs. 11.2 g/dL, and for platelets 88 vs. 126 G/L, respectively. In the cBioPortal cohort, the median values of *NRAS*-mutated and nonmutated patients were for WBC 20.3 vs. 8.8 G/L, for Hb 10.5 vs. 10.8 g/dL, and for platelets 94 vs. 125 G/L, respectively.

**Table 1:** Phenotypic features of ABCMML patients including leukocytosis, anemia, thrombocytopenia, and circulating blasts stratified by the presence or absence of *NRAS* mutations

Parameter	With *NRAS *mutation	Without *NRAS *mutation	*P*-value
WBC ≥ 13 G/L	34/46 (74%)	118/270 (44%)	< 0.001
Hb < 10 g/dL	14/46 (30%)	86/284 (30%)	0.983
PLT < 100 G/L	29/46 (63%)	107/271 (39%)	0.004
PB blasts present	15/38 (39%)	46/227 (20%)	0.013

*WBC* white blood cell counts, *Hb* hemoglobin, *PLT* platelet counts, *PB* peripheral blood

**Table 2:** Phenotypic features of cBioPortal patients including leukocytosis, anemia, thrombocytopenia, and circulating blasts stratified by the presence or absence of *NRAS* mutations

Parameter	With *NRAS *mutation	Without *NRAS *mutation	*P*‑value
WBC ≥ 13 G/L	34/56 (61%)	87/327 (26%)	< 0.001
Hb < 10 g/dL	24/60 (40%)	123/337 (36%)	0.664
PLT < 100 G/L	33/59 (56%)	122/333 (37%)	0.006
PB blasts present	23/48 (48%)	65/285 (23%)	< 0.001

*WBC* white blood cell counts, *Hb* hemoglobin, *PLT* platelet counts, *PB* peripheral blood

## Discussion

In this study we analyzed a national CMML cohort from Austria (ABCMML) and an international cohort of CMML patients (cBioPortal) regarding clinical, epidemiologic, and hematologic features of *NRAS-*mutated patients in order to obtain information on the consistency and general validity of findings.

The clinical outcome of *NRAS*-mutated patients has been reported in a number of different cohorts. In a retrospective analysis of 213 CMML patients seen at the MD Anderson Cancer Center in Texas, 65 patients were analyzed with respect to the presence or absence of *RAS* point mutations (*NRAS* or *KRAS*). The presence of these mutations was not associated with differences in survival times in that study [8]. In a well-characterized cohort of 72 CMML patients from Cleveland, mutations in *NRAS* had no impact on OS [9]. A German CMML cohort of 81 patients was thoroughly molecularly analyzed by NGS [10]. Whereas *TET2*-mutated patients had better overall survival as compared to patients without this mutation, no other molecular parameter, including *NRAS* mutation, revealed any association with survival. In a French cohort of 312 CMML patients, the adverse impact of the *NRAS* mutation was borderline for OS (*p* = 0.06) and significant for AML-free survival (*p* = 0.02) [11]. Finally, in an Italian and Spanish CMML cohort of 214 patients, mutations in *ASXL1, EZH2, NRAS, RUNX1, *and *SETBP1* significantly affected OS [12]. In multivariable COX regression analysis, all these mutations, except *EZH2* mutations, retained an independent prognostic value for OS. Based on these findings, the authors developed a risk score including clinical features and genetic lesions which was validated in an independent cohort consisting of 260 patients diagnosed at the MLL Munich Leukemia Laboratory in Germany. Thus, looking at these data and findings in our study, it seems that the likelihood that *NRAS* mutations will become a significant prognostic factor is dependent on the size of the cohort, since in all four cohorts consisting of more than 200 patients, including the ABCMML, *NRAS* mutations had a significant adverse impact on OS in three of them and borderline significance (*p* = 0.06) in one cohort.

The correlation of phenotypic features with the mutational status in CMML patients has been described previously [19]. Molecular aberrancies in the RAS/MAPK signaling pathway have been shown to be associated with leukocytosis, myeloproliferation, and increased blast cells [20–22]. In both cohorts in our study, it was consistently shown that *NRAS* mutations are associated with an increased white blood cell count and the presence of blast cells in peripheral blood, thus validating previous findings. A new finding is the fact that in both our cohorts, *NRAS*-mutated patients had lower platelet values, a feature that has been reported in CMML patients with mutations in *RUNX1, TET2,* and *SRSF2* [19].

Limitations of this study include the fact that the proportion of patients with leukocytes > 13 G/L was significantly higher in the ABCMML cohort as compared to the cBioPortal cohort. The reason for this imbalance is not completely clear. Increased laboratory screening in recent years among asymptomatic persons may detect some diseases, including CMML, in an earlier phase than in the past. Therefore, older patient series may be enriched in patients with more advanced disease as compared to more recent series. In fact, we have seen a significant drop in the proportion of patients with MP-CMML from 66% to 48% since 2010 in the ABCMML cohort (unpublished data).

Another limitation are changes in the diagnostic criteria of CMML over time since its first description in 1982 of the ABCMML database, suggesting that this patient group is more heterogenous as compared to the cBioPortal group which contains patients who were included over a shorter period of time. Furthermore, it needs to be considered that a proportion of patients in ABCMML, in particular older patients, did not consent to BM puncture. However, we do not believe that this greatly affected diagnostic accuracy, since persistent peripheral blood monocytosis is the most important diagnostic feature, and a genoclinical model has recently been described that uses mutational data, peripheral blood values, and clinical variables to predict the MDS vs. CMML diagnosis with high accuracy in the absence of a BM biopsy result [23]. Moreover, somatic mutations associated with CMML were not only detected in CMML patients confirmed by BM biopsy but also in 57% of patients with nondiagnostic BM features. Interestingly, the OS of mutated patients with non-diagnostic BM was indistinguishable from mutated patients with diagnostic BM suggesting that that the mutational spectrum is a much more sensitive parameter for the detection of myeloid malignancies than BM morphology [24].

In recent years, health care management worldwide has changed from a disease-centered model to a patient-centered model [25]. The adoption of big data characterized by a large amount of digital data which are continually generated by people within clinical care and everyday life will enable the implementation of personalized and precise medicine based on personalized information. Furthermore, these data can be used for validation of findings from national cohorts, and thus make an important contribution to improving patient management.

## Supplementary Information

Suppl Table 1 Patient characteristics in the ABCMML cohort

Suppl Table 2 Patient characteristics in the CMML-cBioPortal cohort

Suppl Table 3 *NRAS* variants and variant allele frequencies in patients of the ABCMML

Suppl Table 4 *NRAS* variants and variant allele frequencies in patients of the cBioPortal
