# Supplementary material for: Characteristics of NRAS-mutated patients with chronic myelomonocytic leukemia in a national (ABCMML) and an international cohort (cBioPortal)
Source: Wien Med Wochenschr. 2025 May 15;175(11-12):267–73. doi: 10.1007/s10354-025-01080-0 (PMC12380971; doi:10.1007/s10354-025-01080-0)
Supplement: Supplementary file 3 — Suppl Table 3 NRAS variants and variant allele frequencies in patients of the ABCMML [file 10354_2025_1080_MOESM3_ESM.docx]

**Suppl Table 3:** *NRAS* variants and variant allele frequencies in patients of the ABCMML

| **ABCMML ID** | **NRAS** | **VAF** |
| --- | --- | --- |
| CMML_1_013 | Y64D | 43 |
| CMML_1_022 | Y64D | 22 |
| CMML_1_032 | G12D | 22 |
| CMML_1_035 | G12D | 47 |
| CMML_1_053 | G12D | 10 |
| CMML_1_105 | G13D | 47 |
| CMML_1_107 | G12D | 6 |
| CMML_1_133 | G13D | 8 |
| CMML_1_137 | G13D | 30 |
| CMML_1_141 | G12D | 37 |
| CMML_1_155 | G12V | 49 |
| CMML_3_174 | A59D | 31 |
| CMML_14_302 | G13N | 9 |
| CMML_16_361 | G13V | 48 |
| CMML_16_362 | G12D | 42 |
| CMML_16_363 | G13V | 6 |
| CMML_16_376 | G13V | 39 |
| CMML_16_379 | G12V | 29 |
| CMML_16-379 | G12V | 34 |
| CMML_1_458 | G12D | 46 |
| CMML_14_476 | Q12N | 47 |
| CMML_1_487 | G12V | 48 |
| CMML_1_574 | G12D | 19 |
| CMML_1_596 | G12D | 6 |
| CMML_1_602 | G12N | 33 |
| CMML_4_605 | Y64N | 35 |
| CMML_18_672 | G12D | 47 |
| CMML_18_675 | G35A | 48 |
| CMML_18_677 | G12D | 48 |
| CMML_1_680 | G12C | 41 |
| CMML_1_700 | G12D | 11 |
| CMML_1_001 | G12D | 48 |
| CMML_1_006 | G12R | 28 |
| CMML_1_033 | Q61K | 30 |
| CMML_1_039 | G12A | 44 |
| CMML_1_042 | G12D | 43 |
| CMML_1_142 | G13V | 38 |
| CMML_1_161 | G12D | 14 |
| CMML_1_171 | G12D | 8 |
| CMML_3_173 | Y64D | 50 |
| CMML_3_175 | G12R | 68 |
| CMML_14_299 | G13V | 41 |
| CMML_16_331 | G12R | 50 |
| CMML_16_332 | G12D | 10 |
| CMML_18_428 | G13D | 36 |
| CMML_3_645 | Q61H | 22 |
