# Supplementary material for: Characteristics of NRAS-mutated patients with chronic myelomonocytic leukemia in a national (ABCMML) and an international cohort (cBioPortal)
Source: Wien Med Wochenschr. 2025 May 15;175(11-12):267–73. doi: 10.1007/s10354-025-01080-0 (PMC12380971; doi:10.1007/s10354-025-01080-0)
Supplement: Supplementary file 4 — Suppl Table 4 NRAS variants and variant allele frequencies in patients of the cBioPortal [file 10354_2025_1080_MOESM4_ESM.docx]

**Suppl Table 4:** *NRAS* variants and variant allele frequencies in patients of the BIOPORTAL

| **BIOPORTAL ID** | **NRAS** | **VAF** |
| --- | --- | --- |
| E-H-100026 | G12D | 32 |
| E-H-100086 | G12D | 33 |
| E-H-100111 | G12S | 39 |
| E-H-100132 | G12D | 2 |
| E-H-100160 | G12D | 36 |
| E-H-100176 | G12R | 24 |
| E-H-100188 | G12V | 48 |
| E-H-100194 | G12D | 6 |
| E-H-100244 | G12D | 14 |
| E-H-100250 | G12D | 47 |
| E-H-100317 | G12D | 6 |
| E-H-100339 | G13D | 44 |
| E-H-102606 | G13D | 9 |
| E-H-102971 | Q61H | 43 |
| E-H-103025 | G12S | 35 |
| E-H-103027 | A146T | 34 |
| E-H-103056 | G12D | 49 |
| E-H-103092 | G13D | 10 |
| E-H-105467 | G12S | 45 |
| E-H-105496 | G12V | 40 |
| E-H-105531 | Q61R | 5 |
| E-H-105696 | G12S | 12 |
| E-H-105712 | G13C | 45 |
| E-H-105834 | G12D | 37 |
| E-H-105896 | Q61P | 20 |
| E-H-105939 | G60E | 42 |
| E-H-105945 | G12S | 10 |
| E-H-105988 | G12V | 42 |
| E-H-110402 | G13V | 21 |
| E-H-110410 | G12S | 14 |
| E-H-110698 | Q61P | 43 |
| E-H-110719 | G13D | 5 |
| E-H-110759 | G12D | 2 |
| E-H-110852 | G12D | 45 |
| E-H-116342 | G12D | 36 |
| E-H-116385 | Q61H | 24 |
| E-H-116458 | G12D | 41 |
| E-H-116481 | G13D | 7 |
| E-H-116483 | G12D | 44 |
| E-H-116508 | G13D | 3 |
| E-H-116598 | G12V | 45 |
| E-H-117175 | A59H | 35 |
| E-H-117178 | G12V | 45 |
| E-H-117969 | G12D | 21 |
| E-H-117974 | G12D | 19 |
| E-H-118057 | G12D | 44 |
| E-H-118171 | Y64D | 25 |
| E-H-118204 | G12S | 3 |
| E-H-118777 | Q61R | 19 |
| E-H-118779 | G12S | 36 |
| E-H-120840 | G12D | 42 |
| E-H-120904 | G12C | 8 |
| E-H-121122 | G12R | 40 |
| E-H-121147 | G12D | 23 |
| E-H-121173 | G12D | 5 |
| E-H-122294 | G12D | 23 |
| E-H-131501 | G12D | 44 |
| E-H-131544 | G12D | 15 |
| E-H-131816 | G12D | 19 |
| E-H-131839 | G12V | 44 |
